# Tumor and Plasma Met Levels in Non-Metastatic Prostate Cancer

**DOI:** 10.1371/journal.pone.0157130

**Published:** 2016-06-14

**Authors:** Deborah R. Kaye, Peter A. Pinto, Fabiola Cecchi, Joseph Reilly, Alice Semerjian, Daniel C. Rabe, Gopal Gupta, Peter L. Choyke, Donald P. Bottaro

**Affiliations:** 1 Urologic Oncology Branch, Center for Cancer Research, National Cancer Institute, Bethesda, MD, 20892, United States of America; 2 Molecular Imaging Program, Center for Cancer Research, National Cancer Institute, Bethesda, MD, 20892, United States of America; Innsbruck Medical University, AUSTRIA

## Abstract

**Objective:**

To measure Met protein content in prostate biopsies guided by fused magnetic resonance and ultrasound imaging, and to measure soluble Met (sMet) protein concentration in plasma samples from patients presenting evidence of prostate cancer.

**Patients and Methods:**

345 patients had plasma samples drawn prior to image-guided biopsy of the prostate. Of these, 32% had benign biopsies. Of the 236 that were positive for prostate adenocarcinoma (PCa), 132 treated by total prostatectomy had Gleason scores of 6 (17%), 7, (55%), 8 (16%), or 9–10 (12%). 23% had evidence of local invasion. Plasma samples were also obtained from 80 healthy volunteers. Tissue Met and plasma sMet were measured by two-site immunoassay; values were compared among clinically defined groups using non-parametric statistical tests to determine significant differences or correlations.

**Results:**

PCa tumor Met correlated significantly with plasma sMet, but median values were similar among benign and malignant groups. Median plasma sMet values were also similar among those groups, although both medians were significantly above normal. Median Met content in primary PCa tumors and sMet concentrations were independent of Gleason score, final pathologic stage and age.

**Conclusion:**

Plasma sMet is not predictive of PCa or its severity in patients with organ-confined or locally invasive disease. Quantitative analysis of Met protein content and activation state in PCa tumor biopsy samples was highly feasible and may have value in follow-up to genomic and/or transcriptomic-based screens that show evidence of oncogenically relevant *MET* gene features that occur at relatively low frequency in non-metastatic PCa.

## Introduction

Prostate cancer (PCa) is the second most common cancer in men and second most common cause of cancer deaths of men in the United States [1]. Over the last two decades, serum prostate specific antigen (PSA) levels have been used to screen for PCa, to predict disease recurrence after therapy, and to predict metastasis; however, the value of this test for screening and outcome prediction remains controversial [2]. Discordance between PSA level and PCa includes cases where patients with metastatic disease have undetectable PSA levels [3], patients with elevated PSA levels post-treatment who never develop metastatic disease [4], and patients for whom PSA values either do not directly correlate with, or are inversely correlated with, disease progression [4]. Although elevated PSA levels resulting from architectural distortion and low levels due to impaired PSA secretion by tumor cells explain some of these discrepancies [5, 6], concerns about the reliability of PSA for monitoring disease progression provide a strong rationale for identifying alternative markers of disease progression, particularly those that are therapeutically targetable.

The hepatocyte growth factor (HGF) receptor tyrosine kinase, known as Met, is overactivated, overproduced and/or mutated in a wide spectrum of prevalent malignancies [7]. Evidence of HGF/Met signaling in PCa is growing. In prostatectomy specimens, Met protein abundance as determined by immunohistochemical (IHC) staining correlated directly with progressive disease [8] and Met positivity was found in a clear majority of [8, 9], or in all of [10, 11], bone metastases samples examined. Consistent with these observations, Met expression in cultured PCa-derived cell lines was found to be inversely related to androgen receptor expression [8, 12], and phase II clinical trials of cabozantinib, a multikinase inhibitor with potent activity against Met, have reported frequent resolution of bone metastases in PCa patients with advanced disease [13–18]. At least 25 experimental therapeutic agents targeting the HGF/Met pathway have been or are currently being tested in over 200 human clinical trials, and the urgent need for biomarkers of oncogenic HGF signaling is becoming widely recognized [7]. HGF concentration was reported to be significantly elevated in the serum of metastatic PCa patients when compared with patients with localized cancer [19], elevated plasma HGF correlated with decreased survival for patients with hormone refractory metastatic PCa [20], and plasma HGF was found to be an independent predictor of PCa metastasis to lymph nodes and disease recurrence after surgery [21]. Our prior studies of another potential surrogate of HGF signaling, soluble Met ectodomain (sMet), showed that sMet levels in plasma and urine correlated directly with burden of PCa tumor xenografts in mice [22], and that urinary sMet was significantly higher in metastatic PCa patients relative to patients with organ-confined disease or controls not known to have any form of cancer [23].

The present study extends our prior work by adapting the two-site electrochemiluminescent sMet immunoassay for use on detergent extracts prepared from flash frozen primary PCa tumor biopsies, and correlates these measurements with PCa stage, grade, Gleason score and plasma sMet levels. Since the advent of PSA screening, the pathologic diagnosis of PCa has been based on the use of systematic trans-rectal ultrasound guided biopsies. Advances in multiparametric magnetic resonance imaging (MRI) have allowed high quality visualization of the prostate and can aid in identification of prostate cancer lesions. We have previously reported on the use of a novel MRI and ultrasound (US) fusion biopsy system for the targeted detection of lesions detected on MRI [24, 25]. Taking advantage of this system allowed more efficient sampling of primary PCa tumor tissue to determine absolute Met protein abundance normalized to total sample protein content.

## Materials and Methods

### Patients and Samples

From 2009 to 2012, primary prostate tumor biopsy specimens and plasma samples were collected from patients who underwent MRI-ultrasound fusion guided prostate biopsy and from patients who proceeded to have radical prostatectomy at the National Cancer Institute (NCI). All patients were enrolled in a NCI Center for Cancer Research (CCR) Institutional Review Board (IRB) approved tissue procurement protocol (NCI-97-C-0147) at the National Institutes of Health (NIH) Clinical Center by informed written consent. Consent documentation records are maintained by the NCI/CCR/IRB and the NIH Clinical Center Office of Protocol Services for a minimum of three years after the completion of the research. Tissue specimens were obtained by MRI-ultrasound fusion guided biopsy using a method reported previously [24, 25]. Serial biopsies from selected sites provided specimens for pathology and protein analysis; the latter were flash frozen on dry ice within 2 minutes of procurement and stored at 80°C prior to analysis. Met and phosphoMet (pMet) protein abundance were measured for biopsy samples selected on the basis of total protein content needed for repeated analysis (14 malignant and 8 benign samples). Tumor tissue samples from 16 patients with pathologically confirmed renal cell carcinoma of various histologies and enrolled in a NCI IRB approved tissue procurement protocol were obtained intraoperatively and processed and stored similarly. Plasma samples were obtained by conventional methods prior to prostatectomy using EDTA as anticoagulant and stored at 80°C prior to analysis. Only patients with a final pathological determination of prostate cancer stage (in those who underwent prostatectomy) and Gleason score were included in the malignant group; all others not known to have any other malignancy were included in the benign group. Plasma samples were also obtained from 80 healthy male volunteers through the NIH Healthy Volunteers program.

### Analysis of Tissue Met and Plasma sMet

Met, phosphotyrosyl-Met, and soluble Met ectodomain (sMet) proteins were measured in tissue and plasma samples as described previously [22]. Briefly, streptavidin-coated 96-well plates were blocked, washed with PBS and coated with a biotin-tagged, affinity-purified human Met ectodomain-specific capture antibody (BAF358, R&D Systems). Wells were washed again before adding sample or sMet standard (358-MT, R&D Systems) for 1 h with shaking. After washing with PBS, detection antibody (AF276, R&D Systems) labeled with MSD-Sulfo-Tag (Meso Scale Discovery, Gaithersburg, MD) was added for 1 h with shaking. Wells were washed with PBS before adding read buffer and plates were read in a Meso Scale Discovery SectorImager 2400. Tissue samples were extracted with ice-cold detergent containing buffer with physical disruption with 0.5mm glass beads and shaking in a bead beater (Mini Bead Beater 8, BioSpec Products, Inc., Bartlesville, OK) as described previously [26]. Assays were performed blinded to study endpoint.

### Statistical Analyses

Absolute plasma sMet concentration and tissue Met content (corrected to total extracted protein) were compared among groups defined by final pathologic stage (in those who underwent prostatectomy) and Gleason score. Statistical analyses were performed using JMP v10.0.2 (SAS Institute Inc.), Excel 2008 for Mac (Microsoft Inc.) and Prism v6.0f (GraphPad Software Inc.) software packages. Wilcoxon, Mann Whitney and Kruskal-Wallis tests, in conjunction with the Bonferroni multiple test correction where necessary, were used to determine statistical differences between groups. Receiver Operating Curves (ROC) were calculated and Area Under the Curve (AUC) determined using JMP. Cutoff values determined using a 45° line drawn tangential to the ROC curve were used to develop specificity, sensitivity and prediction values. Reverse-phase protein array (RPPA) and clinical data for a cohort of 352 cases in the Prostate Adenocarcinoma (PRAD) TCGA Provisional data set were obtained from the M.D. Anderson Reverse Phase Protein Array Core. RPPA scores for Met and phosphoMet (pMet) content were analyzed for relationships with T stage, Gleason score and PSA using Prism v6.0f (GraphPad Software Inc.) software. Within the 352 case cohort, Gleason score was available for 352, clinical T stage data for 348, PSA data for 318, clinical N stage data for 309 and clinical M stage data for 327. Of the latter, all but one case were M0, so correlative tests related distant metastases were not performed.

## Results and Discussion

**Fig 1:**
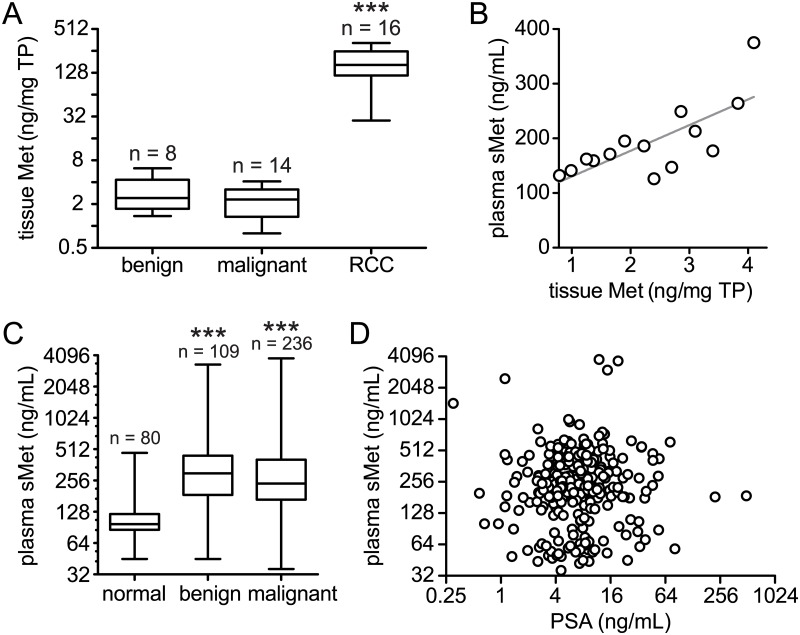
**A.** Box and whisker plots of tissue Met content (ng Met/mg total protein) in primary prostate adenocarcinoma biopsies obtained using ultrasound/MRI fusion image guidance for benign (n = 8) and malignant (n = 14) groups, selected on the basis of total protein content needed for repeated analysis; median values were not significantly different (Mann-Whitney p = 0.4927). The median Met content of a group of renal cell carcinoma (RCC; n = 16) samples of various histologies was significantly higher than either the benign or malignant PCa groups (***, Mann-Whitney p < 0.0001). **B.** Plasma sMet concentrations (ng/ml) and tissue Met content for patients in the malignant PCa group were significantly correlated (Wilcoxon Matched-pairs Two-tailed Signed Rank Test p = 0.0001; Spearman r = 0.7143, p = 0.0027). **C.** Box and whisker plots of plasma sMet (ng/ml) show the median value for a group of healthy male volunteers with no known cancer (normal, n = 80) is significantly lower than either benign (n = 109) or malignant (n = 236) groups (***Spearman r = 0.2939, p < 0.0001). **D.** Matched plasma sMet (ng/ml) vs PSA (ng/ml) values for 274 patients for whom samples for both tests were drawn at the same time show no significant correlation (Spearman r = 0.05492, p = 0.3652).

Demographic information, medical history, relevant laboratory values and pathology data were obtained for each patient. As shown in Table 1, a total of 345 patients, ages 38–60 yrs, were biopsied; 236 were positive for prostate adenocarcinoma (malignant group), 132 of whom were treated by total prostatectomy. Patients with negative biopsy results (109) with no other known cancer were considered benign. Total prostatectomy specimens from the malignant group ranged in Gleason score from 3+3 = 6 (22/17%) to a group with Gleason score totals of 9 or 10 (16/12%). Local invasion and/or regional lymph node involvement was found in 30 patients (23%) and distant metastases in 7 (5%). Median tissue Met content measured in 8 patients with negative biopsy and 14 with positive biopsy were not significantly different (Mann-Whitney p = 0.4927; Fig 1A). Both median values were significantly lower than the median value obtained for another prevalent cancer of the urinary tract, renal cell carcinoma (RCC; Kruskal-Wallis p < 0.0001; Fig 1A). These findings are consistent with the relative frequencies of *MET* gene amplification (determined using GISTIC) and/or mRNA overexpression (2-fold or greater as determined by RNA Seq V2 RSEM) obtained using the cBioPortal [27, 28] to analyze the provisional prostate adenocarcinoma (PRAD) TCGA data set (22/486 cases or 4.5%) *vs*. clear cell RCC (53/531 cases or 10%). Phosphotyrosyl-Met content was undetectable in these tissue samples, which is likely to reflect minimal receptor activation, since maximal receptor activation stimulated *in vitro* by the addition of ATP to several of these samples was readily detected (data not shown). Plasma sMet concentration was significantly correlated with primary tumor Met content in the 14 malignant PCa cases (Spearman r = 0.7143, p = 0.0001; Fig 1B), consistent with a relationship between Met shedding and malignancy reported previously [22].

**Table 1:** Clinical/pathological features of patient group studied.

Clinical/Pathological Feature	Number of Patients (%)
Total Number of Patients Biopsied/Treated	345
Median Age (Range)	60 yrs (38–81)
Negative Targeted Biopsy (benign)	109 (32%)
Positive Biopsy (malignant)	236 (68%)
Total Prostatectomy Specimen	132
Gleason 6	22 (17%)
Gleason 7	73 (55%)
Gleason 8	21 (16%)
Gleason 9 or 10	16 (12%)
Organ Confined	95 (72%)
Local Invasion, Regional Lymph Nodes	30 (23%)
Metastatic	7 (5%)

As for tissue Met, median plasma sMet values in benign (n = 109) and malignant (n = 236) patient groups were not significantly different (Mann-Whitney p = 0.0575); however, both were significantly higher than the median of a healthy volunteer group (normal; n = 80; Spearman r = 0.2939, p < 0.0001; Fig 1C). Receiver Operating Characteristic (ROC) analysis of malignant vs. normal groups using a threshold value of 146 ng/ml yielded 79% sensitivity and 94% specificity values (AUC = 08309, p < 0.0001), but without understanding the basis of elevated sMet levels in benign patients, plasma sMet level appears to have no predictive value for PCa malignancy. A relevant common feature among all patients presenting to the NCI for PCa diagnosis and management is elevated PSA. The median PSA value in the benign cohort was 4.65 ng/ml, significantly above the widely accepted 4.0 ng/ml threshold for prostate biopsy (Wilcoxon Signed Rank Test two-tailed p < 0.0001). No correlation was found, however, between plasma sMet concentration and PSA concentration among 274 patients (including benign and malignant cases) whose samples for both tests were drawn at the same time (Spearman r = 0.05492, p = 0.3652; Fig 1D). In addition, no significant differences or trends in median sMet concentrations were found on the basis of Gleason score, organ-confined disease, locally invasive disease, or metastasis (Table 2). No correlations were found between plasma sMet *vs*. age or pathological grade or stage (data not shown).

**Table 2:** Group plasma sMet medians, ranges, 95% confidence intervals and comparison to normals (shaded) by Wilcoxon Signed Rank Test.

Group	Plasma sMet (ng/ml)		Median 95% Confidence Interval			Wilcoxon Signed Rank Test	
	Median	Range	Actual % Confidence	Lower limit	Upper limit	W^1^	P value^3^
Healthy Volunteer	98	45–471	97	90	106		
Benign	300	45–3341	97	249	367	5735	< 0.0001
Malignant	239	36–3839	96	221	278	26191	< 0.0001
Prostatectomy Specimen	224	36–3034	96	199	253	7720	< 0.0001
Gleason 6	272	69–577	98	177	372	249	< 0.0001
Gleason 7	208	36–951	97	171	259	2249	< 0.0001
Gleason 8	224	42–534	97	179	323	201	0.0001
Gleason 9–10	257	58–425	98	107	426	120	0.0008
Organ confined	222	36–737	96	180	260	4133	< 0.0001
Local invasion^2^	239	44–3034	96	199	323	415	< 0.0001
Metastatic	189	58–633	98	58	633	22	0.0781

^1^Sum of signed ranks

^2^Includes regional lymph node involvement

^3^Two-tailed

**Fig 2:**
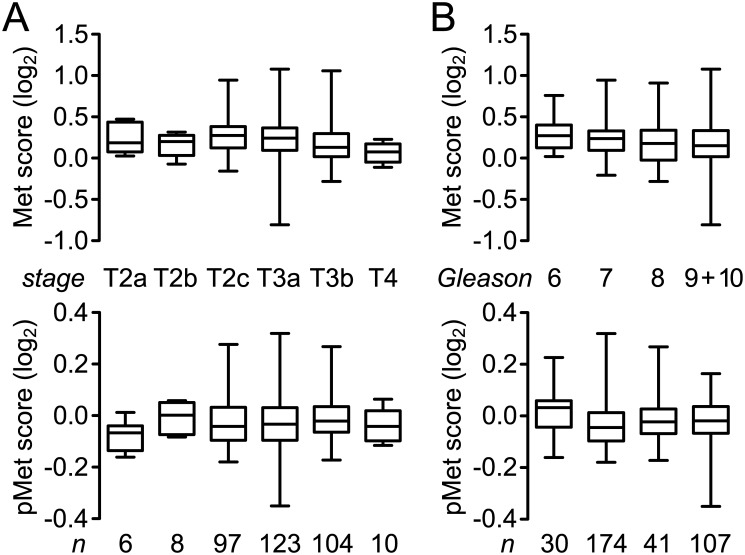
**A.** Box and whisker plots of Met (upper panel) and pMet content (lower panel; RPPA score, log_2_) across clinical T stage subgroups of primary prostate adenocarcinoma biopsies that were obtained as part of the PRAD TCGA Provisional data set. A statistically significant inverse correlation between Met content and higher T stage was found (Table 3). **B.** Box and whisker plots of Met (upper panel) and pMet content (lower panel; RPPA score, log_2_) across clinical Gleason score subgroups of primary prostate adenocarcinoma biopsies that were obtained as part of the PRAD TCGA Provisional data set. A statistically significant inverse correlation between Met content and higher Gleason score was found (Table 3).

Reverse-phase protein array (RPPA) analysis results for Met content in a subset (352) of primary tumor samples in the PRAD TCGA data set were negatively correlated with Gleason score (p = 0.0061; Table 3 and Fig 2B), clinical T stage (p = 0.0050; Table 3 and Fig 2A) and clinical N stage (p = 0.0102, Table 3). These findings are consistent with the statistically non-significant trends we observed in sMet values among T/N stage or Gleason subgroups (not shown). RPPA values for pMet content were not significantly correlated with either Gleason score or T/N stage (Table 3 and Fig 2). Met and pMet scores were also not related to one another or to PSA (not shown). In these contexts the PRAD TCGA RPPA data for Met and pMet are generally consistent with the findings of our patient study.

**Table 3:** Spearman correlation analysis of Met content, pMet content, Gleason score and clinical T and N stage for patient tumor samples^1^ in the prostate adenocarcinoma (PRAD) TCGA Provisional dataset.

Parameter	Met^2^ vs Gleason^3^	pMet^2^ vs Gleason^3^	Met vs T stage^4^	pMet vs T stage^4^	Met vs N stage^5^	pMet vs N stage^5^
Number of XY Pairs	352	352	348	348	309	309
Spearman r	-0.1458	0.03617	-0.1503	0.07766	-0.1460	0.08555
95% confidence interval	-0.2495 to -0.03875	-0.07173 to 0.1432	-0.2544 to -0.04278	-0.03084 to 0.1843	-0.2565 to -0.03163	-0.02962 to 0.1985
P value (two-tailed)	0.0061	0.4988	0.0050	0.1483	0.0102	0.1335

^1^All tissue samples in the PRAD TCGA RPPA Provisional cohort (n = 352) are primary prostate tumor samples

^2^Content scores determined by RPPA analysis

^3^N values for each Gleason score subgroup, from 6 to 10, are shown in Fig 2B

^4^N values for each T stage subgroup, from T2a to T4, are shown in Fig 2A; stage data for 4 cases was unavailable

^5^The N0 subgroup contained 246 cases, the N1 subgroup, 62 cases

## Conclusions

Plasma sMet concentration is not predictive of PCa or its severity in patients with organ-confined or locally invasive disease. Analysis of Met protein content and activation state in primary PCa tumor samples obtained by image-guided biopsy as described here was highly feasible and quantitative. The low incidence of *MET* gene amplification, mutation and/or overexpression in primary prostate tumor samples analyzed by TCGA suggests that Met protein quantitation and activation status may have value in follow-up to genomic and/or transcriptomic-based screens that show evidence of these oncogenically relevant features, to more directly guide therapeutic strategy and for pharmacodynamic evaluation of Met-targeted therapeutics. We believe these findings have value in (1) informing others compelled to do similar work so that resources are used optimally; (2) showing the feasibility and precision of the methods and measurements made in tissue samples obtained with MRI/US-fusion guidance; and (3) improving our understanding of extent and nature of HGF/Met signaling pathway involvement in localized prostate cancer.
